# The level of hypotension during hemorrhagic shock is a major determinant of the post-resuscitation systemic inflammatory response: an experimental study

**DOI:** 10.1186/1472-6793-8-15

**Published:** 2008-07-18

**Authors:** Emmanuel E Douzinas, Ilias Andrianakis, Olga Livaditi, Pantelis Paneris, Marios Tasoulis, Aimilia Pelekanou, Alex Betrosian, Evangelos J Giamarellos-Bourboulis

**Affiliations:** 1 3rd Department of Critical Care Medicine, University of Athens, Medical School, Greece; 2 4th Department of Internal Medicine, University of Athens, Medical School, Greece

## Abstract

**Background:**

To evaluate whether the level of hypotension during hemorrhagic shock may influence the oxidative and inflammatory responses developed during post-ischemic resuscitation.

**Methods:**

Fifteen rabbits were equally allocated into three groups: sham-operated (group sham); bled within 30 minutes to mean arterial pressure (MAP) of 40 mmHg (group shock-40); bled within 30 minutes to MAP of 30 mmHg (group shock-30). Shock was maintained for 60 min. Resuscitation was performed by reinfusing shed blood with two volumes of Ringer's lactate and blood was sampled for estimation of serum levels aminotransferases, creatinine, TNF-α, IL-1β, IL-6, malondialdehyde (MDA) and total antioxidant status (TAS) and for the determination of oxidative burst of polymorhonuclears (PMNs) and mononuclear cells (MCs).

**Results:**

Serum AST of group shock-30 was higher than that of group shock-40 at 60 and 120 minutes after start of resuscitation; serum creatinine of group shock-30 was higher than group shock-40 at 120 minutes. Measured cytokines, MDA and cellular oxidative burst of groups, shock-40 and shock-30 were higher than group sham within the first 60 minutes after start of resuscitation. Serum concentrations of IL-1β, IL-6 and TNF-α of group shock-30 were higher than group shock-40 at 120 minutes (p < 0.05). No differences were found between two groups regarding serum MDA and TAS and oxidative burst on PMNs and MCs but both groups were different to group sham.

**Conclusion:**

The level of hypotension is a major determinant of the severity of hepatic and renal dysfunction and of the inflammatory response arising during post-ischemic hemorrhagic shock resuscitation. These findings deserve further evaluation in the clinical setting.

## Background

Hemorrhagic shock is conceived as an insult frequently leading to systemic inflammatory response syndrome (SIRS), organ damage and multiple-organ dysfunction [1]. The mechanism of pathogenesis of SIRS in the field of hemorrhagic shock is complex and a variety of mechanisms are implicated. The most widely recognized mechanisms are ischemia and reperfusion and stimulation of cells of the innate immune system [2]. Ischemia and reperfusion is mainly participating in oxidative stress and SIRS arising during post-ischemic resuscitation.

Hemorrhagic shock/resuscitation can be viewed as a global ischemia/reperfusion injury insult [3]. The extent of tissue ischemia, that defines the degree of oxygen debt, correlates with a systemic inflammatory response that renders the injured patient at risk for post-resuscitation multiple organ failure (MOF) [4]. Tissue ischemia is determined by the magnitude of the hemorrhagic shock (duration × depth) [5].

Regarding the duration of hemorrhagic shock it has been shown that the longer the shock persists the more intense is the inflammatory response that follows [6]. Similarly, survival seems to improve with early resuscitation and mortality was high with delayed resuscitation and similar to that of unresuscitated animals [7].

To our knowledge, the effect of the depth of hypotension on the oxidative and inflammatory responses at the post-resuscitation period has not been so far systematically explored. However, it has been shown that the depth of shock is a more important factor than the duration of shock in generating a higher mesenteric lymph flow at the post-shock period. Likewise, this lymph engenders greater bioactivity as measured by human polymorhonuclears (PMN) priming for respiratory burst [5]. On the basis of this rationale, a particularly severe shock of even a short duration, can account for an intense SIRS and/or early organ dysfunction that may follow, despite its prompt and vigorous management.

The aim of the present study therefore was to evaluate whether the level of hypotension during hemorrhagic shock may influence SIRS developing during post-ischemic reperfusion. An experimental model in rabbits was designed.

## Methods

### Animals

The study was approved by the Veterinary Directorate of the Prefecture of Athens according to Greek legislation in conformity with the 160/1991 Council Directive of the EU. A total of 15 adult male New Zealand white rabbits of 3.0 to 3.4 kg body weight were used, fasted overnight with access to water *ad libitum*. They were pre-medicated with ketamine (35 mg/kg) and xylazine (5–10 mg/kg) intramuscularly.

### Study design

A marginal ear vein was cannulated, tracheostomy was performed and mechanical ventilation was instituted on a volume mode using a Siemens 900 respirator. Using a tidal volume of 8 mL/kg the frequency was adjusted to maintain PaCO_2 _at 33–37 mmHg. A mixture of air and oxygen was administered to keep PaO_2 _at 95–105 mmHg. Anesthesia was maintained using the aforementioned doses of ketamine and xylazine given intramuscularly every 90 minutes.

Left and right carotid arteries and left internal jugular vein were catheterized for blood pressure monitoring, blood withdrawal for shock induction, and re-infusion of shed blood for shock resuscitation, respectively. The central body temperature was kept between 38° and 39°C with the aid of an electrical blanket.

A stabilization period of 30 min followed the termination of the experimental set-up. Animals were randomly assigned to one of the following groups:

• Group sham (n = 5), animals which underwent all procedures of the experimental setup with the exception of shock; they were similarly mechanically ventilated during the entire experiment while Ringer's lactate was given intravenously at a rate of 0.2 ml/kg per minute

• Group shock-40 (n = 5), induction of shock by withdrawing blood from the left carotid in aliquots of 1.5 mL per min in order to reduce the mean arterial pressure (MAP) to 40 mmHg over the next 30 minutes. MAP was maintained at 40 mmHg over the next 60 minutes by withdrawing or re-injecting blood as required.

• Group shock-30 (n = 5), induction of shock by withdrawing blood from the left carotid in aliquots of 2.0 mL per min in order to reduce the mean arterial pressure (MAP) to 30 mmHg over the next 30 minutes. MAP was maintained at 30 mmHg over the next 60 minutes by withdrawing or re-injecting blood as required.

Heparinized syringes with the shed blood were put on a horizontal rotator at 37°C at 170 rpm. The amounts of blood loss and of Ringer's lactate given were recorded. After the 60 minutes of shock, resuscitation begun by infusing Ringer's lactate twice volume of blood loss plus the shed blood by two different pumps into the left internal jugular vein over the same period as the induction of shock i.e. 30 minutes. FiO_2 _was 0.21–0.28 to maintain PaO_2 _to 90–100 mmHg. In all animals, 2 mL of blood was collected after venipuncture of their ear vein under aseptic conditions. Since the maximal inflammatory [6] and oxidative [8] reaction seems to occur within 2-hour post-resuscitation in most studies, sampling was performed at the beginning of the induction of shock, at the end of shock and at 30, 60, 90 and 120 minutes after the onset of resuscitation and were substituted with double volume of Ringer's lactate. Then animals were sacrificed to avoid their suffering by an intravenous lethal dose of sodium thiopental.

### Laboratory techniques

The serum levels of alanine aminotranferase (ALT), aspartate aminotransferase (AST) and creatinine were estimated by standard techniques (ABBOTT, Chicago, USA). Those of tumor necrosis factor-alpha (TNF-α), interleukin-1beta (IL-1β) and IL-6 were measured by a rabbit enzyme-linked immunosorbent assay. The reagents were provided by the National Institute for Biological Standards and Control (NIBSC, Hertfordshire, UK).

For the estimation of endotoxins (LPS), serum samples were diluted 1:10 in sterile and pyrogen-free water (BioWhitaker, Walkersville, Maryland, USA) and incubated for five minutes at 70°C. The concentration of LPS was then measured by the kinetic QCL-1000 Limulus Amoebocyte Lysate assay (BioWhitaker, lower limit of detection 0.05 EU/mL) using a standard curve created by known concentrations of LPS by *Escherichia coli *serotype O111:B4. All determinations were performed in duplicate and the mean of two observations was applied.

Lipid peroxidation in serum was estimated by the concentration of MDA, as already described [9]. Briefly, a 0.1 mL aliquot of each sample was mixed to 0.9 mL of trichloroacetic acid 20% (Merck, Darmstadt, Germany) and centrifuged at 12,000 g and 4°C for 10 minutes. The supernatant was removed and incubated with 2 mL of thiobarbituric acid 0.2% (Merck) for 60 minutes at 90°C. After centrifugation, a volume of 10 μL of the supernatant was injected into a high-performance liquid chromatography system (HPLC, Agilent 1100 Series, Waldbronn, Germany) with the following characteristics of elution: Zorbax Eclipse XDB-C18 (4.6 × 150 mm, 5 μm) column under 37°C; mobile phase consisting by a 50 mM K_3_PO_4 _(pH: 6.8) buffer and methanol 99% at a 60/40 ratio with a flow rate of 1 mL/min; fluorometric detection with signals of excitation at 515 nm and emission at 535 nm. The retention time of MDA was 3.5 minutes and it was estimated as μmol/mL by a standard curve created with 1,1,3,3-tetramethoxy-propane (Merck). All determinations were performed in duplicate.

Total antioxidant status (TAS) was assessed in all serum samples by a colorimetric assay based on the decrease of the optical density of the blank produced by each sample in analogy to its antioxidant property (Randox Laboratories, San Francisco, CA, USA). Optical density was read at 600 nm (Hitachi Spectrophotometer, Tokyo, Japan). All determinations were done in duplicate and results were expressed as μmol/L.

Oxidative burst of polymorphonuclear cells (PMNs) and mononuclear cells (MCs) was assessed as described by others [10]. Aliquots of 100 μL of whole blood were incubated with 2 μL of dichlorohydrofluoresceine diacetate (DDF-DA) (Molecular Probes, Oregon, USA) (0.5 mM) for 15 min at 37°C in a humidified atmosphere containing 5% CO_2_. DDF-DA is oxidized in cell cytoplasm by reactive oxygen species to 2, 7,-dichrorofluoresceine (DCF), which is a highly fluorescent compound (excitation 515 nm, emission 545 nm). Samples undergoing the same procedure but not loaded with DDF-DA, served as negative controls for the determination of the baseline level. Samples were analyzed by the FACSCalibur flow cytometer (BDBiosciences, SanJose, CA). Two gates were set on mononuclear cells (MCs) and polymorphonuclear cells (PMNs). For each measurement, at least 10000 cells were acquired by forward and side-scatter characteristics. Results were expressed as geometric mean of the fluorescence intensity (GMFI) for gated cell populations.

### Statistical analysis

Results were expressed as means ± SD or medians and range depending on the normal or non-normal distribution of values. Comparisons between groups were performed by Mann-Whitney U test with post-hoc Bonferroni analysis. Any value of p below 0.05 was considered statistically significant.

## Results

The mean ± SE volume of Ringer's lactate administered was 72 ± 5 and 76 ± 7 mL in shock-40 and shock-30 groups, respectively (NS).

Serum levels of AST, ALT and creatinine of the three study groups are given in Table 1. Serum AST of group shock-30 was higher than that of group shock-40 at 60 and 120 minutes after start of resuscitation (p of comparisons between groups <0.05). The same applied for serum creatinine at 120 minutes (p < 0.05 between groups).

**Table 1:** Serum biochemical parameters.

	Group sham	Group shock-40	Group shock-30
	AST (Mean ± SD, U/L)		
Baseline	18.0 ± 1.9	25.0 ± 9.7^a^	21.7 ± 9.3^c, e^
End of shock	20.4 ± 2.2	25.4 ± 8.9^a^	29.3 ± 7.2^c, e^
30 min	20.8 ± 3.1	29.0 ± 8.4^a^	37.0 ± 5.0^c, e^
60 min	24.0 ± 1.6	32.6 ± 9.1^a^	44.0 ± 2.6^c, f^
90 min	23.4 ± 3.2	39.0 ± 13.9^b^	54.7 ± 6.0^d, e^
120 min	24.6 ± 2.7	45.8 ± 15.4^b^	61.0 ± 6.6^d, f^

	ALT (Mean ± SD, U/L)		
Baseline	17.6 ± 3.2	20.2 ± 7.4^a^	17.7 ± 4.2^c, e^
End of shock	19.6 ± 3.6	23.4 ± 7.5^a^	20.3 ± 8.5^c, e^
30 min	18.4 ± 2.5	25.0 ± 7.2^a^	23.0 ± 8.5^c, e^
60 min	18.8 ± 2.6	28.4 ± 8.3^a^	24.3 ± 8.1^c, e^
90 min	18.2 ± 1.6	31.0 ± 8.9^a^	24.6 ± 8.1^c, e^
120 min	19.4 ± 1.5	34.4 ± 8.5^b^	31.7 ± 8.1^c, e^

	Creatinine (Mean ± SD, mg/dl)		
Baseline	1.10 ± 0.42	0.90 ± 0.07^a^	1.10 ± 0.20^c, e^
End of shock	1.08 ± 0.38	0.96 ± 0.15^a^	1.33 ± 0.32^c, e^
30 min	1.04 ± 0.34	1.02 ± 0.08^a^	1.27 ± 0.40^c, e^
60 min	0.94 ± 0.21	1.08 ± 0.13^a^	1.37 ± 0.35^c, e^
90 min	0.88 ± 0.28	1.08 ± 0.22^a^	1.50 ± 0.36^d, e^
120 min	0.90 ± 0.29	1.22 ± 0.32^a^	1.63 ± 0.46^d, f^

Serum concentrations of aspartate aminotransferase (AST), alanine aminotransferase (AST) and creatinine of animals undergoing sham operation (group sham); animals undergoing hemorrhagic shock to MAP 40 mmHg (group shock-40) before start of resuscitation; and animals undergoing hemorrhagic shock to MAP 30 mmHg (group shock-30) before start of resuscitation.

Statistical comparisons after Bonferroni correction

Group sham vs group shock-40: ^a^pNS (non-significant); ^b^p < 0.05

Group sham vs group shock-30: ^c^pNS (non-significant); ^d^p < 0.05

Group shock-40 vs group shock-30: ^e^pNS (non-significant); ^f^p < 0.05

**Figure 1:**
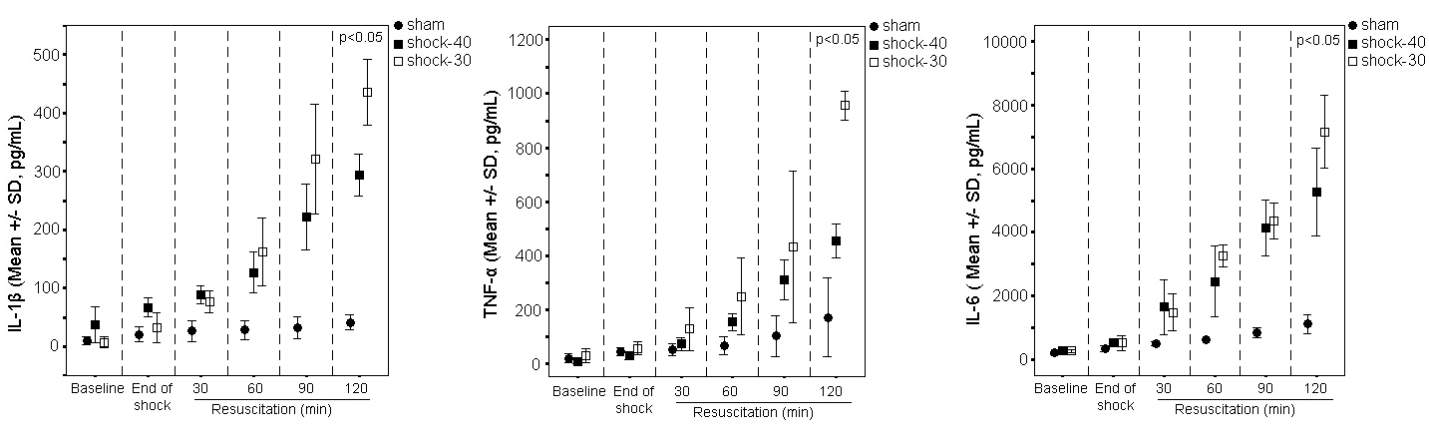
**Cytokines levels in all study groups**. Concentrations of interleukin (IL)-1β (left panel), tumor necrosis factor-alpha (TNF-α) (middle panel) and IL-6 (right panel) in serum of animals undergoing sham operation (group sham); animals undergoing hemorrhagic shock to MAP 40 mmHg (group shock-40); and animals undergoing hemorrhagic shock to MAP 30 mmHg (group shock-30). P values refer to comparisons between group shock-40 and group shock-30 at the respective time interval.

Serum concentrations of IL-1β, IL-6 and TNF-α are given in Figure 1. IL-1β of group shock-40 was significantly higher than group sham at 30, 60, 90 and 120 minutes after start of resuscitation (p of comparisons between groups <0.05 in all cases). Similar differences were found between group sham and group shock-30. A difference between groups shock-40 and shock-30 was noted only at 120 minutes after start of resuscitation were IL-1β of group shock-30 was greater than serum IL-1β of group shock-40 (p < 0.05 between groups). Similar differences among the three studied groups were recorded both for serum IL-6 and TNF-α.

**Figure 2:**
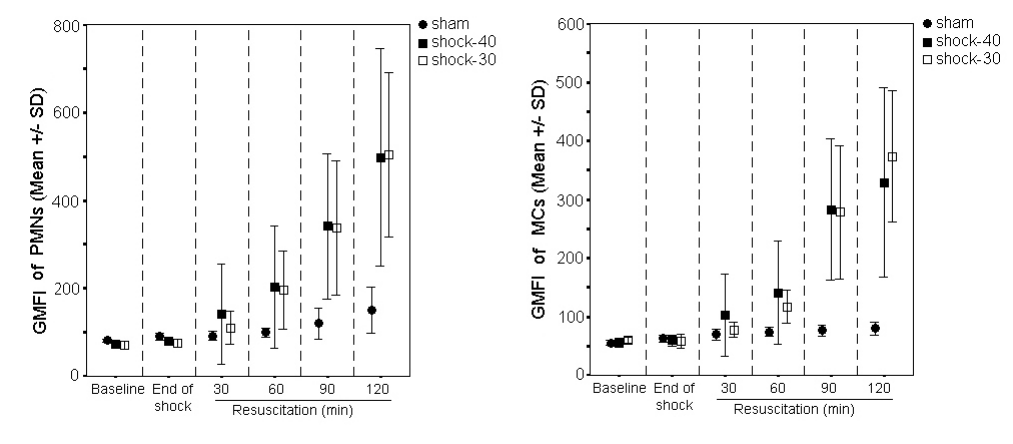
**Oxidative burst in all study groups**. Oxidative burst of polymorphonuclears (PMNs) and mononuclear cells (MCs) of animals undergoing sham operation (group sham); animals undergoing hemorrhagic shock to MAP 40 mmHg (group shock-40); and animals undergoing hemorrhagic shock to MAP 30 mmHg (group shock-30). GMFI, geometric mean of the fluorescence intensity.

Oxidative burst on PMNs and MCs are shown in Figure 2. This was statistically higher in group shock-40 than group sham on both PMNs and MCs at 60, 90 and 120 after start of resuscitation (p < 0.05 between groups at each time interval). Similar findings were also noted between group sham and group shock-30. However no difference was detected between shock-40 and shock-30 groups.

Serum levels of MDA, TAS and LPS are shown in Table 2. No differences were found between shock-40 and shock-30 groups.

**Table 2:** Serum oxidative parameters and endotoxin.

	Group sham	Group shock-40	Group shock-30
	MDA (Mean ± SD, μmol/mL)		
Baseline	0.61 ± 0.16	0.81 ± 0.15^a^	0.69 ± 0.07^c, e^
End of shock	0.88 ± 0.09	1.77 ± 0.09^b^	1.69 ± 0.04^d, e^
30 min	1.04 ± 0.27	2.32 ± 0.12^b^	2.16 ± 0.08^d, e^
60 min	1.14 ± 0.34	2.61 ± 0.19^b^	2.65 ± 0.06^d, e^
90 min	1.26 ± 0.40	2.85 ± 0.22^b^	2.79 ± 0.67^d, e^
120 min	1.36 ± 0.49	3.23 ± 0.19^b^	3.20 ± 0.09^d, e^

	TAS (Mean ± SD, μmol/L)		
Baseline	0.59 ± 0.11	0.47 ± 0.14^a^	0.39 ± 0.08^c, e^
End of shock	0.70 ± 0.12	0.80 ± 0.19^a^	0.75 ± 0.13^c, e^
30 min	0.73 ± 0.11	0.86 ± 0.18^a^	0.77 ± 0.09^c, e^
60 min	0.77 ± 0.10	0.75 ± 0.16^a^	0.61 ± 0.13^c, e^
90 min	0.79 ± 0.11	0.59 ± 0.08^b^	0.55 ± 0.10^d, e^
120 min	0.77 ± 0.08	0.49 ± 0.01^b^	0.47 ± 0.08^d, e^

	LPS (Median-range, EU/mL)		
Baseline	0.26 (0.05–0.56)	0.39 (0.05–4.18)^a^	0.05 (0.05–1.38)^c, e^
End of shock	0.10 (0.05–0.13)	0.57 (0.13–1.65)^b^	0.05 (0.05–0.85)^c, e^
30 min	0.05 (0.05–0.17)	0.32 (0.05–0.56)^a^	0.05 (0.05–0.96)^c, e^
60 min	0.05 (0.05–0.48)	0.24 (0.06–0.49)^a^	0.14 (0.05–0.89)^c, e^
90 min	0.05 (0.05–0.54)	0.60 (0.05–1.23)^a^	0.05 (0.05–1.45)^c, e^
120 min	0.05 (0.05–0.46)	0.42 (0.05–1.58)^a^	0.05 (0.05–8.38)^c, e^

Serum concentrations of malndialdehyde (MDA), total antioxidant status (TAS) and endotoxin (LPS) of animals undergoing sham operation (group sham); animals undergoing hemorrhagic shock to MAP 40 mmHg (group shock-40); and animals undergoing hemorrhagic shock to MAP 30 mmHg (group shock-30).

Statistical comparisons after Bonferroni correction

Group sham vs group shock-40: ^a^pNS (non-significant); ^b^p < 0.05

Group sham vs group shock-30: ^c^pNS (non-significant); ^d^p < 0.05

Group shock-40 vs group shock-30: ^e^pNS (non-significant); ^f^p < 0.05

## Discussion

Hemorrhagic shock is a situation characterized by severe ischemia of perfused tissues. Start of reperfusion of the ischemic tissues may lead to SIRS, a process that many authors conceive as a result of the abundant generation of oxygen free radical species by the reperfused tissues [2]. However, no data exist in the literature for the role of shock per se to the contribution of post- reperfusion SIRS. To elucidate that, an animal model of hemorrhagic shock was designed in rabbits. Animals were separately bled either to MAP equal to 40 mm Hg or to MAP equal to 30 mm Hg. Serum kinetics of pro-inflammatory mediators were compared between groups following resuscitation.

Results revealed that reperfusion after shock was accompanied by hepatocellular injury and renal dysfunction evidenced by the rise of AST and creatinine serum levels, respectively (Table 1). Both AST and creatinine were higher among animals shocked at 30 mm Hg than animals shocked at 40 mm Hg revealing a propensity of shock at lower blood pressure for more severe organ dysfunction. In animals shocked at 40 mmHg, serum creatinine failed to increase above the values of sham-operated animals. This was not the case with animals shocked at 30 mmHg where serum creatinine started to increase within the first 90 minutes after start of resuscitation.

On the same content, in all shocked animals serum pro-inflammatory cytokines as well as serum MDA and cellular oxidative burst was increased. This was accompanied by a reciprocal decrease of serum TAS (Figures 1 and 2 and Table 2). These findings are consistent with the concept of ischemia and reperfusion injury arising through the generation of reactive oxygen species. However, pro-inflammatory cytokines were higher among animals previously shocked to MAP 30 mm Hg than those shocked to MAP 40 mm Hg (Figure 1), indicating that inflammation intensified along with the depth of arterial pressure during hemorrhagic shock.

However, leukocyte oxidative burst was similar (Figure 2) between the two groups as if it had already attained its maximal response at MAP 40 mmHg. Although is likely that the depth of shock does not accordingly intensify the oxidative response, there are reasons that introduce uncertainty on this assumption: (a) in fact, ROS formation rate might be higher in group shock-30 but since these were consumed at the sites of oxidative process they maintained similar blood levels with group shock-40; in that case however, MDA should become higher with reciprocal lower TAS levels in group shock-30, which is not the case in the current model (Table 2); (b) there are potential limitations regarding the assays for the range of the actual molecules' concentration leading to a lack of difference; the assays, however, seem to detect reliably the concentrations of molecules, since their increase follows the progress of resuscitation (Table 2, Figure 2).

In fact, the extension in the resuscitation period might reveal a difference in the oxidative response between the two groups. If not, the data may suggest that other factors, in addition to oxidative stress, contribute to the exacerbation of the inflammation and the organ injury that are observed with more severe hypotension during hemorrhagic shock. Such a factor could be the rate of hemorrhage that was higher in the shock-30 than shock-40 animals to achieve the indicated difference of blood pressure in the same period of time. The higher rate of hemorrhage leading to acute hemorrhagic shock of particularly low arterial pressure may significantly contribute to the subsequent inflammatory response elicited through the early activation of NF-kB even in the absence of resuscitation procedures [11].

Many authors have suggested that bacterial translocation from the gut may participate in the pathogenesis of post-resuscitation SIRS [12,13]. Implication however of the above process is unlikely since serum LPS did not differ between groups in the described setting (Table 2).

A former study of our group revealed that serum of animals subjected to post-ischemic resuscitation may stimulate the release of pro-inflammatory cytokines by U937 monocytes through a p38-MAP kinase-dependent mechanism [14]. It may be postulated that the factor circulating in serum creating the difference in serum kinetics of TNFα, IL-1β and IL-6 in the present study may act though the p38-MAP kinase pathway. This hypothesis is consistent with recent data by Frink et al [15]. The authors have shown that cytokine production by Kupffer cells following trauma-hemorrhage is mediated through the toll-like receptor-4 (TLR4). Stimulation of TLR4 is accompanied by intracellular activation of the p38-MAP kinase.

The administration of Ringer's lactate as repletion fluid may represents a limitation of the study since it has been implicated in causing inflammatory reaction [16,17]. However, it appears unlikely that Ringer's lactate was responsible for the observed difference of inflammatory response seen, because the volumes administered between the groups were similar. Although in general, resuscitation results in greater proinflammatory gene transcription than no resuscitation [16], Ringer's lactate has been shown to possess similar effects to both colloids [16] and normal saline [18] on indices of inflammation.

## Conclusion

The presented results show that the degree of hypotension determines the inflammatory reaction and the severity of hepatic and renal dysfunction that arise during post-ischemic hemorrhagic shock resuscitation. Similar data are important for establishing the mechanistic link between the level of arterial blood pressure during shock and distant organ injury and for providing evidence in the continuing debate of the optimal resuscitation of post injury hemorrhagic shock.

## Abbreviations

ALT: alanine aminotransferase; AST: aspartate aminotransferase; IL-1β: interleukin-1beta; IL-6: interleukin-6; LPS: lipopolysaccharide; MDA: malondialdehyde; MAP: mean arterial pressure; SIRS: systemic inflammatory response syndrome; TAS: total antioxidant status; TNF-α: tumour necrosis factor-alpha

## Authors' contributions

EED designed the study, analyzed the results and participated in the writing of the manuscript. IA performed animal experiments, participated in the analysis of results. OL performed estimation of ALT, AST, LPS, cytokines, MDA, TAS and cellular oxidative burst. PP performed animal experiments. MT performed animal experiments. AP performed estimation of LPS. AB participated in the analysis of results and editing of the manuscript. EJG–B participated in analysis of the data and in writing of the manuscript. All authors read and approved the final manuscript.
